# The antihyperlipidemic effect of a combined supplement of standardized dry extracts of amla (*Emblica officinalis*), walnut (*Juglans regia*), olive (*Olea europaea*) and red yeast rice (*Monascus purpureus*) powder: Reduction in circulatory low-density lipoprotein-cholesterol (LDL-C) and remnant cholesterol (RC) levels in patients with hypercholesterolemia

**DOI:** 10.3389/fphar.2023.1280234

**Published:** 2023-11-27

**Authors:** Michel P. Hermans, Yvan Dierckxsens, Isabelle Janssens, Laurence Seidel, Adelin Albert, Sylvie A. Ahn, Michel F. Rousseau, Amjad Khan

**Affiliations:** ^1^ Division of Endocrinology and Nutrition, Cliniques Universitaires St-Luc and Institut de Recherche Expérimentale et Clinique (IREC), Université Catholique de Louvain, Brussels, Belgium; ^2^ Laboratoire Tilman, Baillonville, Belgium; ^3^ B-STAT, Centre Hospitalier Universitaire, Liège, Belgium; ^4^ Division of Cardiology, Cliniques Universitaires St-Luc and Pôle de Recherche Cardiovasculaire, Institut de Recherche Expérimentale et Clinique (IREC), Université Catholique de Louvain, Brussels, Belgium; ^5^ Nuffield Division of Clinical Laboratory Sciences, Radcliffe Department of Medicine, University of Oxford, Oxford, United Kingdom; ^6^ Department of Biochemistry, Liaquat University of Medical and Health Sciences, Jamshoro, Pakistan

**Keywords:** Cholesfytol NG^®^, hyperlipidemia, LDL-C, remnant cholesterol, amla, red yeast rice, olive, walnut

## Abstract

**Background:** Hyperlipidemia is associated with a higher rate of cardiovascular, cerebrovascular, and peripheral vascular disease. Conventional drugs such as statins are effective in controlling hyperlipidemia; however, they are associated with various side effects, especially myalgia. Nutraceutical lipid-lowering interventions are becoming increasingly popular, particularly among patients who are intolerant or refractory to statins. Substantial preclinical and clinical evidence suggests that extracts of amla, walnut, and olive, and red yeast rice (RYR) powder possess significant antihyperlipidemic effects.

**Aims:** This study aimed to evaluate the efficacy, safety, and patient satisfaction of a combined supplementation of standardized dry extracts of amla fruit (500 mg), walnut leaves (50 mg), olive fruit (25 mg), and RYR powder (33.6 mg) (Cholesfytol NG®) in hypercholesterolemic patients.

**Methods:** This was a *real-life setting*, retrospective, observational, single-arm, non-randomized study in hypercholesterolemic patients (total cholesterol (TC) ≥ 200 mg/dL or low-density lipoprotein-cholesterol (LDL-C) ≥ 130 mg/dL), enrolled at 57 general practitioner (GP) surgeries in Belgium from March 2020 to January 2022. These patients received a GP-prescribed daily single dosage of two oral tablets of Cholesfytol NG® supplementation for 2 months to overcome their hypercholesterolemia in the absence of a conventional lipid-lowering drug (n = 208) or with a lipid-lowering drug (n = 13). At 2-month follow-up, the lipid profile was re-evaluated, alongside a patient’s questionnaire on treatment general satisfaction and willingness to pursue supplementation.

**Results:** After supplementation, TC decreased by 15%, LDL-C by 19%, non-high-density lipoprotein-cholesterol (non-HDL-C) by 20% (all *p* < 0.0001), triglycerides (TG) by 9% (*p* = 0.0028) (−18.4%, *p* = 0.0042, in patients with baseline TG > 180 mg/dL, n = 58), and remnant cholesterol (RC) by 12% (*p* = 0.0001). These changes were unaffected by statin intolerance status in patients who received Cholesfytol NG® alongside statin. The supplement was well tolerated by all patients, and no serious adverse events or supplement-emergent effects were reported. Most patients were satisfied with the supplementation and wanted to pursue the nutraceutical.

**Conclusion:** According to the results of this study, a combined supplementation of amla, walnut, and olive extracts, and RYR powder exerts a significant antihyperlipidemic effect, leading to a decrease in circulatory LDL-C and RC levels in patients with hypercholesterolemia. The supplementation bears excellent safety and tolerability, and is rated as satisfactory and pursuable, even among patients with statin intolerance.

Clinical Trial Registration: clinicaltrials.gov; identifier number: NCT06002893

## 1 Introduction

Hypercholesterolemia is defined as elevated blood levels of total cholesterol (TC) and/or low-density lipoprotein-cholesterol (LDL-C, also known as ‘bad’ cholesterol) or non-high-density lipoprotein-cholesterol (non-HDL-C, all the ‘bad’ cholesterol or TC burden) and is diagnosed by a blood lipid profile test. Hypercholesterolemia is among the leading modifiable risk factors of atherosclerotic cardiovascular disease (ASCVD) including cerebrovascular disease, coronary heart disease, peripheral arterial disease, and aortic disease (Keys, 1980; Castelli et al., 1986; Lacoste et al., 1995; Stamler, 1996), especially in at-risk patients, such as those with pre‐existing atherosclerotic disease, hypertension, impaired plasma glucose, and low high-density lipoprotein-cholesterol (HDL‐C, also known as ‘good’ cholesterol) levels, diabetes, older age, and cigarette smoking. In addition, high blood plasma triglyceride (TG) levels add another independent risk factor for the development of coronary artery disease (CAD) and cardiovascular events (Collaboration et al., 2004; Sarwar et al., 2007; Nordestgaard and Varbo, 2014). Hypercholesterolemia is generally asymptomatic until substantial atherosclerosis has developed. Complications of hypercholesterolemia and atherosclerosis are diverse and include sudden cardiac arrest, myocardial infarction, ischemic stroke, ischemic cardiomyopathy, acute limb ischemia, claudication, and erectile dysfunction. Among the key risk factors of secondary hypercholesterolemia are excessive consumption of saturated fats, trans-fatty acids, cholesterol-rich diets, and a sedentary lifestyle. Other associations include obesity, diabetes, hypothyroidism, nephrotic syndrome, and cholestasis.

Treatment of hypercholesterolemia typically involves lifestyle modifications such as nutritional changes (adopting low-cholesterol and low-saturated fat food diet), regular physical activity/exercise, smoking cessation, weight loss, and conventional drug treatment including 3-hydroxy-3-methyl glutaryl-CoA (HMG-CoA) reductase inhibitors (statins), selective cholesterol absorption inhibitors (SCAIs), bile acid sequestrants, proprotein convertase subtilisin/kexin type 9 (PCSK9) inhibitors, adenosine triphosphate-citrate lyase (ACL) inhibitors, nicotinic acid, fibric acid derivatives, and omega-3 fatty acids and fatty acid esters (Authors/Task Force Members ESC Committee for Practice Guidelines (CPG) ESC National Cardiac Societies, 2019). Among these medications, statins have proven to be most effective in reducing LDL-C levels and decreasing ASCVD risk by 15%–37%, and they are recommended as a first-line therapy for the primary and secondary prevention of ASCVD. In patients with high CV risk, to achieve additional LDL-C reduction and reduce ASCVD risk, the current guidelines for cholesterol management recommend two drugs, Ezetimibe (a SCAI) or a PCSK9 inhibitor (Alirocumab and Evolocumab), alongside statin (Choi and Na, 2019). However, besides LDL-C, there is also a substantial residual CV risk driven by elevated levels of remnant cholesterol (RC). Remnant cholesterol, defined as the cholesterol content of all non-LDL and non-HDL particles, has been recognized as an individual cardiovascular risk factor, independent of LDL-C, particularly in patients with metabolic disorders (Chait et al., 2020; Ginsberg et al., 2021). Extensive studies have revealed that RC levels are substantially linked with a greater risk of CAD as well as major cardiovascular adverse incidences (Castañer et al., 2020; Zhao et al., 2020; Shao et al., 2022), even when LDL-C is at goal on effective statin (±Ezetimibe) treatment (Varbo et al., 2014; Sandesara et al., 2019; Castañer et al., 2020; Ginsberg et al., 2021; Quispe et al., 2021; Doi et al., 2023).

Although conventional lipid-lowering drugs (LLDs) can significantly decrease LDL-C levels, these medications have diverse side effects (Feingold, 2021); for instance, statins can cause muscle aches, tenderness or weakness (myalgia), joint pain, liver injury, increase in blood sugar, nausea, indigestion, and upset stomach. Statin-associated muscle symptoms (SAMS) are clinically the adverse side effects experienced by over 70% of patients, resulting in discontinuation of statin therapy. Statin intolerance has been implicated with an increased risk of recurrent cardiac incidents. Other LLDs such as Ezetimibe can cause dizziness, upset stomach, and diarrhea; side effects of fibrates include indigestion, abdominal pain, and fatigue; bile acid resins can cause constipation, bloating, stomach pain, and nausea. Niacin consumption can cause flushing, itching, increased heart rate, and heartburn, while omega-3 fatty acids can lead to taste disturbances, “fish burps,” indigestion, and upset stomach.

**FIGURE 1:**
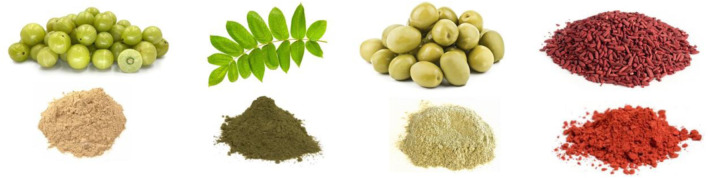
New-generation Cholesfytol NG® is a dietary supplement consisting of a combination of (L to R) standardised dry extracts of amla fruit (500 mg), walnut leaves (50 mg), olive fruit (25 mg, equivalent to 5 mg hydroxytyrosol), and RYR (33.6 mg, equivalent to 1.45 mg of monacolines) powder, which produces a synergistic antihypercholesterolemic effect arising from the biologically active chemical constituents of the extracts.

Indeed, as a first-line approach, adopting lifestyle changes such as optimized nutrition aimed to reduce modifiable risk factors at the individual level, is an effective strategy in the management of lifestyle-related health conditions. In recent years, there has been a growing scientific and clinical interest in the therapeutic properties of functional nutritional supplements, both in the prevention and management of lifestyle-related health conditions such as hyperlipidemia, low HDL-C level, abdominal obesity, hyperglycemia, and hypertension (Vanhoutte, 2009; Suganya et al., 2016). Specifically, for the management of hyperlipidemia, among the several botanicals with demonstrated biological activities, the extracts of *Emblica officinalis* Gaertn. *or Phyllanthus emblica L*. fruit, commonly known as Indian gooseberry or amla, *Juglans regia L*. (walnut) leaves, *Olea europaea L*. (olive) fruit, and *Monascus purpureus* (red yeast rice or RYR) powder have emerged as an effective antihyperlipidemic agents with an excellent safety and tolerability profile (Figure 1).

### 1.1 Amla extract as natural medicine

For centuries, in Ayurveda, amla has been utilised for diverse health conditions including CAD and metabolic disorder conditions such as hypertension, type 2 diabetes, and obesity. Modern pharmacological studies have demonstrated antioxidant, antihyperglycemic, antihyperlipidemic, and anti-inflammatory therapeutic effects of amla, both in human and animal model studies (Yokozawa et al., 2007; Antony et al., 2008a; Akhtar et al., 2011; Gul et al., 2022). Amla reportedly contains a wide range of compounds, including quercetin, corilagin, phyllanemblinins, ellagic acid, chebulic acid, emblicanins, phyllaemblic acid, pedunculagin, methyl gallate, punigluconin, chlorogenic acid, gallic acid, and other compounds (Gul et al., 2022).

### 1.2 Walnut extract as natural medicine

Walnut extract has been traditionally utilized for various medicinal purposes including hyperlipidemia, diabetes, and heart diseases (Delaviz et al., 2017; Gupta et al., 2019; Sharma et al., 2022). Current evidence suggests that walnut extract is antioxidative, anti-inflammatory, antidiabetic, antihyperlipidemic, hypolipidemic, antihypertensive, and hepato- and nephro-protective (Delaviz et al., 2017; Gupta et al., 2019; Sharma et al., 2022). Walnut leaves are rich in antioxidants including phenolic acids (largely caffeoylquinic acids) and flavonoids, consisting mainly of juglone (5-hydroxy-1,4-naphthoquinone), quercetin, and its derivatives (Delaviz et al., 2017; Gupta et al., 2019; Sharma et al., 2022).

### 1.3 Olive extract as natural medicine

Olive has been used for centuries as a traditional medicine for multiple health conditions such as hyperglycemia, hypercholesterolemia, hyperuricemia, hypertension, and inflammation (Hashmi et al., 2015; Visioli et al., 2020). Olive extract exhibits a wide array of therapeutic effects including antidyslipidemic**,** antidiabetic, antioxidant, antihypertensive, cardioprotective, and anti-inflammatory (Hashmi et al., 2015; Visioli et al., 2020).

### 1.4 RYR powder as natural medicine

RYR exhibits diverse biological activities including hypolipidemic, antiatherosclerotic, antidiabetic, antiobesity, immunomodulatory, anti-inflammatory, and antihypertensive (Zhu et al., 2019). RYR is a proven effective agent against hypercholesterolemia (Cicero et al., 2019). In terms of its chemical composition, over 100 compounds have been isolated from RYR including monacolins, organic acids, flavonoids, polysaccharides, sterols, pigments, decalin derivatives, and others (Zhu et al., 2019).

In an effort to treat hypercholesterolemia and higher TG levels without side effects in a natural way, an optimized oral dietary supplement consisting of a combination of standardized dry extracts of amla fruit, walnut leaves, olive fruit, and RYR powder has been developed (new-generation Cholesfytol NG®; Tilman s. a., Baillonville, Belgium). The different extract components of this product have documented complementary effects on dyslipidemia and hypercholesterolemia (Bulotta et al., 2014; Hu et al., 2014; Barbagallo et al., 2015; Efentakis et al., 2015; Verhoeven et al., 2015; Sahebkar et al., 2016; Cicero et al., 2017; Tshongo Muhindo et al., 2017; Hermans et al., 2020). According to the reported literature, LDL-C lowering is achieved with RYR monacolin K (MK) (Endo, 1980; Lin et al., 2008; Pérez-Jiménez et al., 2018) and amla PCSK9 inhibition (Variya et al., 2016; Variya et al., 2018). A TG-lowering effect of this nutraceutical is also present, as amla is a natural nuclear receptor agonist of peroxisome proliferator-activated receptor alpha (PPAR-α) (Variya et al., 2016). The walnut leaves extract also lowers TG due to ellagitannins’ hypotriglyceridemic activity *via* an increase in the peroxisomal *β*-oxidation of fatty acids in the liver (Shimoda et al., 2009). Furthermore, amla and olive extracts are strong antioxidants, and both improve endothelial function (Shimoda et al., 2009; Bulotta et al., 2014; Hu et al., 2014; Efentakis et al., 2015).

To date, there have been no prospective real-life studies on the effect of an association of extracts of amla, walnut, olive, and RYR powder on different aspects of the hyperlipidemia including RC in patients with or without a history of statin intolerance. The present *real-life setting*, pragmatic, and observational exploratory study aimed to evaluate the safety and clinical efficacy of general practitioner (GP)-prescribed Cholesfytol NG® supplementation (single dose of two tablets a day for 2 months) on LDL-C, HDL-C, non-HDL-C, and RC in patients with hypercholesterolemia in a Belgian population.

## 2 Materials and methods

### 2.1 Study design and patients

This was a *real-life setting*, retrospective, single-arm non-controlled, non-randomized, and open-label observational clinical study of a random population of hypercholesterolemic patients, enrolled at 57 GP surgeries in Belgium from 19 March 2020 to 31 January 2022. These patients received a GP prescription of Cholesfytol NG® supplement, taken as a single dose of two tablets a day in the evening for 2 months in the absence of an LLD (94.1%) or with an LLD (5.9%). Patients were assessed at the study inclusion visit (T0) and the 2-months follow-up visit (T1) at the GP. As this was an observational study of data collection of patients’ medical records from GPs, ethics approval was not required as per the Belgian law (7 May 2004 Art. 3 §2) regarding research involving human subjects. The study was registered in the clinicaltrials.gov registry (identifier number NCT06002893).

Patients were required to meet the following inclusion criteria: (1) age ≥ 21 years; (2) both male or female; (3) TC ≥ 200 mg/dL; (4) LDL-C ≥ 130 mg/dL; (5) with or without a history of myalgia complaints and/or diabetes; (6) No prior treatment of cholesterol lowering agents and/or patients whose cholesterol-lowering treatment did not allow them to reach LDL-C target; (7) patients who had stopped their cholesterol-lowering treatment because of side effects including myalgia. (8) Cholesfytol NG® 240 supplement prescription as a single dose of 2 tablets a day for 2-months. Exclusion criteria included pregnant or breastfeeding patients.

### 2.2 Cholesfytol NG® supplement

Cholesfytol NG® supplement is a new-generation optimized oral dietary supplement manufactured by Tilman s. a., Baillonville, Belgium, and contains 500 mg of dry extract of amla fruit, standardized to ≥60.0% hydrolyzable tannins, which includes compounds like emblicanin-A, emblicanin-B, punigluconin, and pedunculagin; 50 mg walnut leaves dry extract standardized to ≥0.15% 3-O-caffeoylquinic acid (3-CQA); 25 mg olive fruit dry extract standardized to contain 20% hydroxytyrosol (5 mg); and 33.6 mg of RYR powder (equivalent to 1.45 mg of monacolines).

### 2.3 Variables

The data collected for each patient includes demographics (age and sex), baseline, and 2-months follow-up clinical data: lipids [measured by CardioChek Plus; TC (mg/dL); LDL-C (mg/dL; Friedewald’s formula); HDL-C (mg/dL); TG (mg/dL)]; non-HDL-C (mg/dL) was calculated as [TC *minus* HDL-C]; RC (mg/dL) was calculated as [TC *minus* LDL-C *minus* HDL-C] (Varbo et al., 2014); plasma glucose was measured with CardioChek Plus (mg/dL); systolic and diastolic blood pressure (SBP and DBP); and waist circumference (WC, cm). Patients were asked about their current treatment (or already tried in the case of LLDs), about any intolerance to statins, and the type of LLD they are using (statins, fibrates, Ezetimibe, statin + Ezetimibe, other LLDs including RYR and omega-3 supplements, or other dietary supplements). The use of BP-lowering and glucose-lowering drugs was also recorded. During treatment, the occurrence of SAMS side effects were also documented (Stroes et al., 2015). A general patient questionnaire was also filled in, to assess patient’s general satisfaction and desire to continue Cholesfytol NG® supplementation beyond the study period. All information was recorded in a case report form (CRF).

### 2.4 Statistical analyses

The results are presented as mean and standard deviation (SD) and median and interquartile range (IQR) for quantitative variables and as frequency tables (numbers and percentages) for categorical variables. The association between two quantitative variables was measured by the correlation coefficient. The comparison of the means of the same quantitative variable before and after treatment was performed by a paired-sample *t*-test as well as by the Wilcoxon signed-rank test. To compare the means of the two groups, the *t*-test for unpaired samples or the Kruskal–Wallis test was used. To compare proportions, the chi-squared test or Fisher exact test was used. The mean changes (in pure values and percentages compared to the baseline value) of the different parameters after treatment were also presented with their 95% confidence intervals (95% CI). The analysis of patient satisfaction according to different covariates was studied by ordinal logistic regression. The association between satisfaction and the covariate was quantified by the odds ratio (OR) and its 95% CI. The results were considered significant at the 5% uncertainty level (*p* < 0.05). All statistical calculations were performed on the maximum amount of data present. Missing data were not replaced or imputed. Analyses were performed using SAS® version 9.4 and R version 4.1.1 statistical software.

## 3 Results

### 3.1 Patients’ enrollment

**FIGURE 2:**
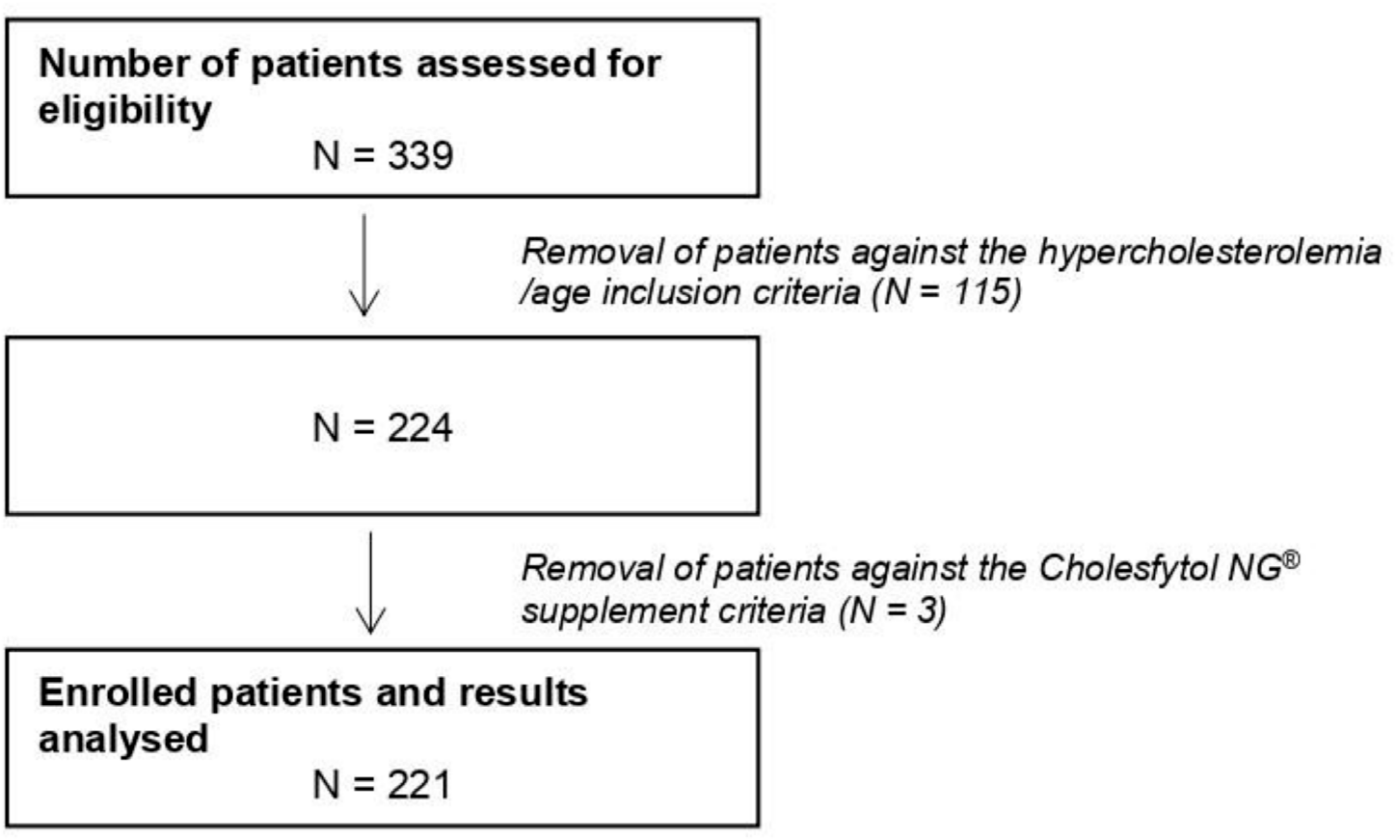
Study consort flow diagram.

Figure 2 shows the study consort flow diagram. In total, 339 patients were assessed for eligibility, out of which 115 patients were excluded due to not fulfilling some of the inclusion criteria (i.e., TC < 200 mg/dL, LDL-C < 130 mg/dL, or age <21 years). Furthermore, three patients were excluded: for two of them, Cholesfytol NG® supplementation was no longer deemed necessary by the GP, while for one patient, the prescription of supplementation was not two tablets per day, leaving the study population to a total of 221 patients. Of these 221 patients, 119 had a minor violation to compliance with the study supplement period, i.e., 8 ± 2 weeks, while the remaining 102 patients complied with the supplement period of 2 months. Table 1 shows the demographics of patients. The mean age of patients was 61 ± 12 years, of whom 39.4% were men.

**TABLE 1:** Demographic characteristics of study patients (N = 221).

Variable	Mean ± SD/number (%)	Median (IQR)	Min–max
Age (years)	61.3 ± 12.3	61.1 (53.7–70.3)	24.3–90.5
Gender (N = 218)			
Women	132 (60.6)		
Men	86 (39.4)		

### 3.2 Patients’ history and treatment for hypercholesterolemia

At the time of supplement prescription/enrollment, among the total 221 patients, 143 (64.7%) had never received or tried an LLD before, 49 (22.1%) had stopped using LDDs, and 29 (13.1%) were still using LLDs. Of these 78 (35.3%) patients, 54 (69.2%) had received one LLD, 19 (24.3%) two LLDs, and five (6.4%) three LLDs. Statins were by far the most predominant LLDs (23%), followed by RYR supplement (12%), while fibrates, Ezetimibe, a combination of statin and Ezetimibe, omega-3 fatty acids, other dietary supplements, and other LLDs barely reached a few percents. There was a history of statin intolerance in 23% of patients, 33% of patients were taking BP-lowering medications, and 5% of patients were taking glucose-lowering drugs. Among the 29 patients who were on LLDs at enrollment, 13 (5.9%) kept their LLDs or were prescribed another LLD (mainly prescription statins and dietary supplements other than RYR and omega-3 fatty acids) together with Cholesfytol NG® supplement, while the remaining 16 (7.2%) patients were switched to Cholesfytol NG® supplement (alone). Overall, a total of 208 (94.1%) patients received Cholesfytol NG® supplement prescription as a single dose of two tablets a day for 2 months, in the absence of an LLD/other supplement for the management of their hypercholesterolemia. The majority of patients were compliant (89.1%) with the supplementation.

### 3.3 Lipid changes between T0 and T1

Table 2 shows the effect of Cholesfytol NG® supplementation on patients’ lipid profile parameters. At T0, the mean lipid values were as follows: TC: 251 ± 33 mg/dL, LDL-C: 163 ± 29 mg/dL, non-HDL-C: 194 ± 34 mg/dL, HDL-C: 58 ± 18 mg/dL, RC: 31 ± 16 mg/dL, and TG: 152 ± 69 mg/dL. At T1, there was a significant decrease in TC [-38 ± 37 mg (relative drop: −15.1%), *p* < 0.0001]. The same was true for LDL-C [-32 ± 31 mg/dL (−19.3%), *p* < 0.0001], non-HDL-C [-38 ± 39 mg/dL (−19.7%), *p* < 0.0001], RC [-4 ± 13 mg/dL (−12.3%), *p* < 0.0001], and TG [-14 ± 67 mg/dL (−9.3%), *p* = 0.0028]. HDL-C remained stable (0.19 ± 11.1 mg/dL, *p* = 0.80). Among the 58 patients with baseline TG > 180 mg/dL, the latter decreased from 234 ± 60 mg/dL to 191 ± 89 mg/dL (−18.4%), *p* = 0.0042). This decrease differed significantly (*p* = 0.0089) from patients with TG ≤ 180 mg/dL at baseline, among whom the relative decrease was 2.5% (NS), non-significant.

**TABLE 2:** Effect of Cholesfytol NG® supplementation on patients’ lipid profile parameters (N = 221).

Lipid parameter	N	T0	T1	Absolute difference	Relative average change (%)
		Mean ± SD	Mean ± SD	Mean ± SD	
		Median (IQR)	Median (IQR)	CI95%	
				*p*-value	
TC (mg/dL)	217	251 ± 32.9	213 ± 32.0	−38.0 ± 36.6	−15.1
		245 (230–265)	212 (194–234)	(-42.9 to −33.1)	
				**<0.0001**	
LDL-C (mg/dL)	213	161 ± 26.0	130 ± 29.8	−31.8 ± 31.1	−19.3
		156 (144–172)	129 (111–145)	(-36.0 to −27.6)	
				**<0.0001**	
Non-HDL-C (mg/dL)	214	193 ± 33.7	155 ± 33.1	−38.0 ± 38.5	−19.7
		187 (171–211)	155 (135–174)	(-43.2 to −32.87)	
				**<0.0001**	
HDL-C (mg/dL)	214	57.8 ± 17.8	58.0 ± 16.3	0.19 ± 11.1	+0.35
		55 (45–67)	57.5 (45–67)	(-1.30–1.68)	
				0.80	
RC (mg/dL)	206	31.0 ± 16.1	27.2 ± 16.7	−3.75 ± 13.4	−12.3
		27.5 (20–38)	24 (16–33)	(-5.59 to −1.91)	
				**<0.0001**	
TG (mg/dL)	209	150 ± 67.6	136 ± 68.3	−14.1 ± 67.1	−9.3
		134 (100–183)	124 (89–161)	(-23.2 to −4.90)	
				**0.0028**	

TC, total cholesterol; LDL-C, low-density lipoprotein-cholesterol; HDL-C, high-density lipoprotein-cholesterol; RC, remnant cholesterol; TGs, triglycerides. *p*-value < 0.05 was considered statistically significant (denoted in bold).

### 3.4 Non-lipid changes between T0 and T1

Table 3 shows the effect of Cholesfytol NG® supplementation on patients’ fasting plasma glycemia, tensor, and biometric parameters. The mean plasma glucose at T0 was 96 ± 18 mg/dL, SBP was 132 ± 14 mmHg, DBP was 79 ± 8 mmHg, and WC was 97 ± 15 cm. At T1, the mean plasma glucose was 94 ± 14 mg/dL, SBP was 128 ± 11 mmHg, DBP was 79 ± 8 mmHg, and WC was 96 ± 15 cm. The mean decrease in glucose was −2.35 ± 14.5 mg/dL (−2.5%), *p* = 0.026. SBP decreased by 4 ± 12 mmHg (−3%), *p* < 0.0001. DBP remained stable (*p* = 0.21). WC decreased by −0.85 ± 3 cm (−1%), *p* = 0.020.

**TABLE 3:** Effect of Cholesfytol NG® supplementation on patients’ fasting plasma glycemia, tensor, and biometric parameters (N = 221).

		T0	T1	Absolute difference	
Variable	N	Mean ± SD	Mean ± SD	Mean ± SD	Relative average change (%)
		Median (IQR)	Median (IQR)	CI95%	
				*p*-value	
Glucidic parameters					
Fasting plasma glycemia (mg/dL)	191	96.4 ± 18.2	94.0 ± 13.5	−2.35 ± 14.5	−2.5
		94 (87–101)	92 (85–101)	(-4.42 to −0.28)	
				**0.026**	
Blood pressure parameters					
Systolic blood pressure (mmHg)	210	132 ± 14.2	128 ± 11.5	−3.86 ± 11.6	−3.0
		130 (120–140)	130 (120–135)	(-5.44 to −2.28)	
				**<0.0001**	
Diastolic blood pressure (mmHg)	210	79.5 ± 8.28	78.9 ± 8.15	−0.62 ± 7.21	−0.75
		80 (75–85)	80 (71–85)	(-1.60 to 0.36)	
				0.21	
Biometrics					
Waist size (cm)	72	97.1 ± 15.8	96.2 ± 15.6	−0.85 ± 3.04	−0.93
		94.5 (89.5–104)	94.0 (87–102)	(-1.57 to −0.14)	
				**0.020**	

*p*-value < 0.05 was considered statistically significant (shown in bold).

### 3.5 Safety and tolerability of supplementation

The supplement was well tolerated, and no adverse events or supplement-associated effects were reported by any of the study participants. During the study period of a median of 71 days (IQR 62–91) of taking the nutraceutical, a small number of patients (6%) reported mild gastrointestinal effects.

### 3.6 *Cholesfytol NG* *®* supplement satisfaction

Overall, most patients (79%) were (very) satisfied with the treatment at the end of follow-up (T1); 86% wanted to continue, and 82% felt an improvement in their condition. The level of patient satisfaction was studied according to demographic characteristics and changes (%) in each metabolic parameter by ordinal logistic regression, with very dissatisfied and dissatisfied patients combined (Table 4). The results showed that the greater the drop in TC (OR = 1.07, *p* < 0.0001), LDL-C (OR = 1.05, *p* < 0.0001), and non-HDL-C (OR = 1.05, *p* < 0.0001), the greater the satisfaction of patients.

**TABLE 4:** Patient’s demographic characteristics and changes (%) in metabolic parameters and supplement satisfaction by ordinal logistic regression at univariate level (N = 221).

	Very satisfied		Satisfied		Neutral		Very dissatisfied/dissatisfied		Univariate modeling					
Variable	N	Number (%)	N	Number (%)	N	Number (%)	N	Number (%)	N		OR		*p*-value	
		Mean ± SD		Mean ± SD		Mean ± SD		Mean ± SD						
Gender	53		114		33		13		213		1.022 (0.61–1.72)		0.93	
Female		35 (66.0)		62 (54.4)		21 (63.6)								
Male		18 (34.0)		52 (45.6)		12 (36.4)								
Age (years)	54	59.3 ± 10.8	116	61.6 ± 12.4	33	63.1 ± 13.1	9	60.3 ± 14.2	216		0.99 (0.97–1.01)		0.23	
Drop in TC (%)	54	19.2 ± 12.5	116	15.5 ± 9.79	33	7.48 ± 13.1	9	2.14 ± 13.9	212		**1.07 (1.04–1.09)**		**<0.0001**	
Drop in LDL-C (%)	53	26.0 ± 16.4	114	20.7 ± 13.2	32	6.15 ± 20.3	9	6.48 ± 15.5	208		**1.05 (1.03–1.07)**		**<0.0001**	
Drop in non-HDL-C (%)	52	26.0 ± 15.1	116	19.3 ± 16.5	33	9.21 ± 5.3	9	3.77 ± 17.1	210		**1.05 (1.03–1.07)**		**<0.0001**	
Increase in HDL-C (%)	52	3.88 ± 17.2	116	2.42 ± 20.8	33	0.20 ± 15.3	9	3.53 ± 14.8	210		1.00 (0.99–1.02)		0.50	
Drop in RC (%)	51	8.22 ± 58.7	110	3.89 ± 60.2	32	17.1 ± 34.2	9	−19.9 ± 51.2	202		1.00 (0.99–1.01)		0.90	
Drop in TG (%)	51	11.6 ± 31.5	112	3.43 ± 33.9	33	2.88 ± 37.1	9	−15.5 ± 47.3	205		1.00 (1.00–1.02)		0.051	
Drop in fasting plasma glycemia (%)	49	−0.04 ± 13.5	106	0.28 ± 27.1	23	2.03 ± 13.9	9	−1.69 ± 13.85	187		0.99 (0.98–1.01)		0.88	
Drop in SBP (%)	54	1.81 ± 8.83	112	3.16 ± 8.12	31	0.47 ± 7.00	8	3.88 ± 8.80	205		1.00 (0.97–1.03)		0.94	
Drop in DBP (%)	54	−1.28 ± 9.30	112	1.51 ± 8.36	31	−2.17 ± 13.1	8	2.67 ± 6.99	205		0.99 (0.97–1.02)		0.63	
Drop in waist circumference (%)	13	1.01 ± 5.75	48	0.81 ± 1.55	7	−0.28 ± 1.25	2	1.72 ± 2.44	70		1.08 (0.90–1.28)		0.41	

TC, total cholesterol; LDL-C, low-density lipoprotein-cholesterol; HDL-C, high-density lipoprotein-cholesterol; RC, remnant cholesterol; TGs, triglycerides; SBP, systolic blood pressure.

DBP, diastolic blood pressure. *p*-value < 0.05 was considered statistically significant (shown in bold).

## 4 Discussion

**FIGURE 3:**
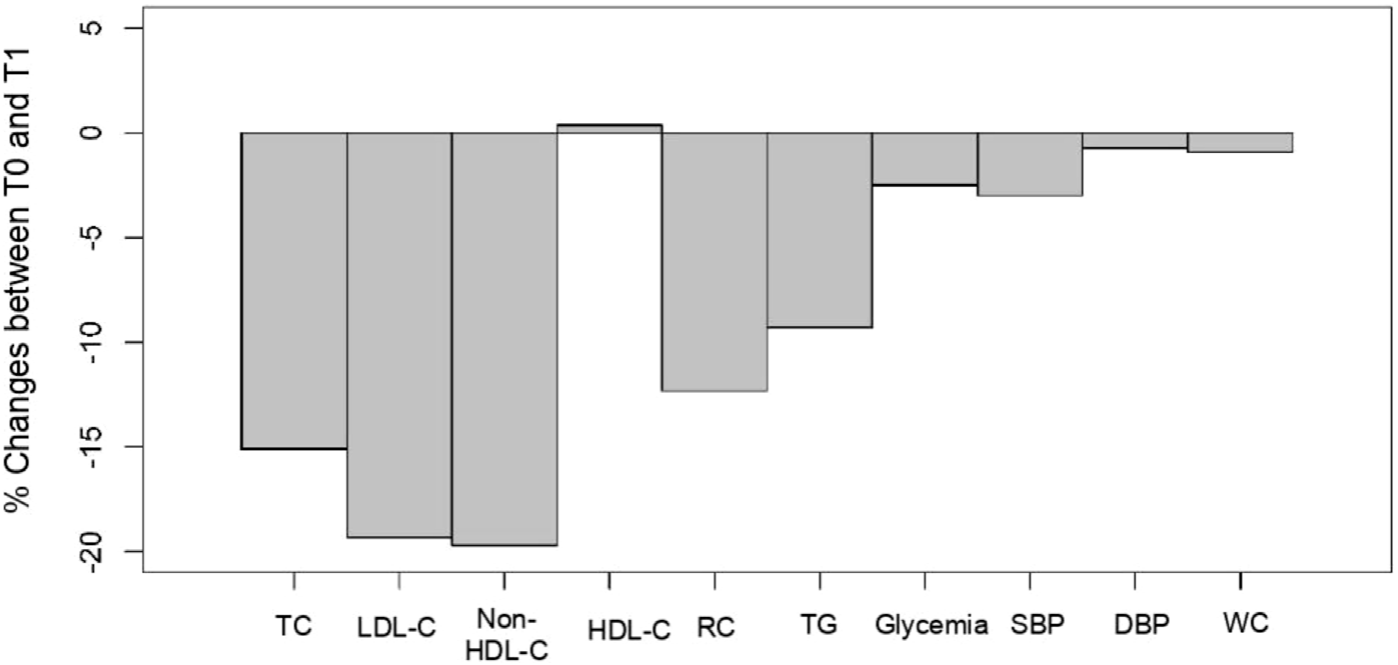
Cholesfytol NG® supplement effect. Mean change (%) in patients’ metabolic parameters between T0 and T1 (N = 221). TC, total cholesterol; LDL-C, low-density lipoprotein-cholesterol; HDL-C, high-density lipoprotein-cholesterol; RC, remnant cholesterol; TGs, triglycerides; SBP, systolic blood pressure; DBP, diastolic blood pressure; and WC, waist circumference.

The main objective of the present exploratory study conducted by GPs in patients (N = 221) suffering from hypercholesterolemia was to evaluate the efficacy and safety of a combined supplement of standardized dry extracts of amla fruit, walnut leaves, olive fruit, and RYR powder, taken as two tablets/day for a period of 2 months, in the absence of conventional LLD treatment (94.1%), and in a small number of patients (5.9%) with statins/another dietary supplement. Figure 3 summarizes the treatment outcomes for these patients. As regards lipid parameters, TC decreased by 15%, LDL-C by 20%, and non-HDL-C by 16% under real-life conditions. It was not accompanied by side effects usually associated with prescription statins (Gopa et al., 2012; Stroes et al., 2015; Dugani et al., 2016). Reductions of such magnitudes are clinically relevant, especially as they are achieved using a nutraceutical with a most satisfactory safety profile (Baigent et al., 2005). Next to the basic lipid profile, the study evaluated the effect of a nutraceutical on RC. At baseline, patients’ mean RC levels exceeded the upper limit of 30 mg/dL, which corresponds to an excess of atherogenic cholesterol unrelated to LDL-C (Varbo et al., 2014; Sandesara et al., 2019; Castañer et al., 2020; Ginsberg et al., 2021; Quispe et al., 2021; Doi et al., 2023). On treatment, the mean RC level decreased by 12.3%, putting it below the pathological threshold of 30 mg/dL. These results show that in addition to lowering the LDL-C cardiovascular risk, this nutraceutical also has a beneficial impact on lowering the residual risk associated with RC.

The supplementation also resulted in a significant decrease of 3% in SBP and 1% in WC (Figure 3). There was also an improvement of 2.5% in patients’ plasma glycemia. In those patients who fully complied with the 2-months supplementation, a drop of 4% and 2% in SBP and DBP, respectively, was observed.

This supplementation may be envisaged to lower LDL-C and RC levels, while also avoiding the risk of side effects, on a long-term basis as an addition in primary prevention settings while also avoiding the risk of side effects, when the distance to LDL-C or distance to RC can be bridged and/or in patients who do not wish to take prescription statins over the long term when baseline cardiovascular risk is low (P et al., 2013). This nutraceutical could also be considered an add-on option for patients in primary or secondary cardiovascular prevention when they do not reach their LDL-C target despite receiving the best standards of care (including statins) and/or when experiencing SAMS on high-intensity statin therapy (Stroes et al., 2015).

The clinically significant reductions in patients’ lipid profile achieved with Cholesfytol NG® supplementation is the result of synergistic antihypercholesterolemic effects arising from the supplement component extracts (active chemical constituents), as reported in previous randomized clinical trials (RCTs). The treatment benefits of amla in hypercholesterolemia and atherosclerosis are well established in animal experiments (Rajak et al., 2004; Yokozawa et al., 2007; Variya et al., 2018) and human studies (Antony et al., 2008a; Antony et al., 2008b; Gopa et al., 2012; Usharani et al., 2013; Sinha et al., 2014; Upadya et al., 2019; Usharani et al., 2019) with excellent safety and tolerability profile. In a recent double-blind, placebo-controlled RCT involving patients with metabolic syndrome, a daily 1,000 mg amla fruit extract supplementation for 12 weeks led to a significant mean % change in LDL-C (−21.8%), TC (−11.11%), TG (−19.22%), and HDL-C (+22.16%) as compared to the placebo group (Usharani et al., 2019). In another study in dyslipidemic patients, the same dosage and duration of amla fruit extract supplementation resulted in a significant reduction in LDL-C, VLDL-C, TC, and TG levels in comparison to the placebo group (Upadya et al., 2019). In a study by Gopa et al., a 500 mg amla extract capsule/day intake for 42 days was non-inferior to 20 mg simvastatin in lowering LDL-C (Gopa et al., 2012). The possible mechanism of action of amla in regulating dyslipidemia is *via* PCSK9-inhibition, PPAR-α agonism (Variya et al., 2016; Variya et al., 2018), as well as its strong antioxidant effects (Antony et al., 2008a; Gul et al., 2022). As compared to the native LDL-C, oxidized LDL-C are considered more harmful to the arterial wall. Oxidized LDL-C are pro-oxidants and can lead to tissue damage and stimulate the development of atherosclerotic lesions (Navab et al., 1996). Overall, there is substantial preclinical and clinical evidence to suggest the antidyslipidemic pharmacological effect of the amla fruit extract.

Red yeast rice is also well known for its hypolipidemic effect, demonstrated by many RCTs (Journoud and Jones, 2004; Becker et al., 2009; Cicero et al., 2019; Wang et al., 2019; Cicero et al., 2021; Li et al., 2022). Currently, RYR is among the most widely utilized dietary supplements for the management of high cholesterol levels in Asian and European countries (Sahebkar et al., 2016). Many clinicians use RYR as a substitute treatment for their hyperlipidemic patients who are intolerant to statin, due to its excellent safety and tolerability profile (Fogacci et al., 2019). RYR has a comparable lipid-lowering activity to statins and lowers the cardiovascular disease risk in patients with statin intolerance or those who do not meet their goals after a single course of treatment with statins or ezetimibe. RYR has been suggested as a treatment option for lowering LDL-C levels and ASCVD risk for people with mild-to-moderate hypercholesterolemia who are ineligible for statin therapy, particularly those who are unable to implement lifestyle modifications, and also for those who are otherwise eligible for statin therapy but unwilling to take a pharmacologic therapy (Cicero et al., 2023). The antihyperlipidemic therapeutic effect of RYR is believed to be due to its principal active constituent monacolins, more specifically monacolin K (2.9 mg from Cholesfytol NG® supplementation). Monacolin K has the same chemical structure as Lovastatin (a type of statin) and is a competitive inhibitor of the cholesterol production pathway’s rate-limiting enzyme HMG-CoA reductase (HMGR) (Endo, 1980; Istvan and Deisenhofer, 2001; Pérez-Jiménez et al., 2018). In a recent meta-analysis study of 20 RCTs consisting of an overall patient population of 6,663, RYR supplementation for a period of 2 months to 2 years was associated with reduction in serum LDL-C levels by 1.02 mmol/L (∼39.4 mg/dL) (with 95% confidence) as compared to the placebo group, exhibiting substantial lipid-lowering efficacy comparable to the treatment effect of low-dose statins (e.g., 10 mg Simvastatin, 20 mg Lovastatin, and 40 mg Pravastatin) (Gerards et al., 2015). The study also reported a decrease in levels of TG, as well as an improvement in HDL-C levels. In another double-blind, placebo-controlled RCT, an RYR supplementation for 16 weeks led to a significant reduction in patients’ serum LDL-C (23.0%) and TC (15.5%) levels as compared to the placebo group (Bogsrud et al., 2010).

Walnut extracts have also been extensively studied for their antihyperlipidemic effect. A recent meta-analysis study of 26 RCTs has shown that supplementation of the walnut diet was associated with improvement in the blood lipid profile without having a negative impact on body weight or blood pressure (Guasch-Ferré et al., 2018). In another meta-analysis study of 13 RCTs by Banel et al., high-walnut-enriched diets significantly decreased LDL-C and TC (Banel and Hu, 2009). A large body of animal model studies also supports the antihyperlipidemic effect of walnut leaves extract supplementation, leading to a significant reduction in TC, LDL-C, VLDL-C very-low-density lipoprotein (VLDL) cholesterol, and TG levels and improvement in HDL-C levels (Mahmoodi et al., 2011; Jelodar et al., 2020; Azh et al., 2021).

The antidyslipidemic and/or cardioprotective properties of olive fruit extract have been extensively investigated in both animal and human studies and attributed to the strong antioxidative effects of its polyphenolic constituents, predominantly hydroxytyrosol or HXT (received as 10 mg from Cholesfytol NG® supplementation) and its derivatives (Raederstorff, 2009). Several RCTs have reported an improvement in the lipid profile and a decrease in oxidized LDL-C levels after an intake of olive diets rich in HXT (Marrugat et al., 2004; Covas et al., 2006; de la Torre-Carbot et al., 2010; Mateos et al., 2016; Fonollá et al., 2021; Cicero et al., 2022). As a matter of fact, the European Food and Safety Agency (EFSA) published a report in 2011 asserting that consuming 5 mg of HXT (or its derivatives) from olives on a daily basis protects LDL particles from oxidative damage (EFSA Panel on Dietetic Products, Nutrition and Allergies (NDA), 2011). Hydroxytyrosol is listed on the US Food and Drug Administration (FDA) GRAS (Generally Recognized as Safe) status list (Nova, 2019), supporting its excellent safety profile.

Besides demonstrating the antihypercholesterolemic efficacy, this study also affirmed the excellent safety and tolerability of Cholesfytol NG® supplementation, thus avoiding the risk of side effects. Although statins are the most commonly used LLDs in clinical practice to treat hypercholesterolemia/LDL-C both in primary and secondary prevention of cardiovascular events, they can sometimes harm muscles, resulting in inflammation and discomfort. Impairment in memory, hyperglycemia, and liver injury are other negative consequences associated with statin therapy (Joseph et al., 2015). These complications pose additional health risks, especially for older patients like those in this study. In 2012, the FDA amended the safety profile of statins to include a warning about the possibility of elevated fasting plasma glucose and HbA1c levels (Feingold, 2021).

We acknowledge that our study has the inherent limitations of an observational, uncontrolled, open-label, and short-term design with a limited patient cohort. Since this was a clinical evaluation in a *real-life setting*, the GPs had complete discretion over which patients the supplementation was prescribed, and the Cholesfytol NG® supplement prescription was not based on a set of predetermined criteria. The absence of a control group makes it impossible to assess possible confounding effects, including lifestyle, over the observation period. Since patients remained in usual care over a relatively short study period and were not given instructions to reinforce lifestyle or change diet, such confounders are unlikely. The sample of mostly Caucasian patients who took the nutraceutical in a compliant manner may not necessarily be superimposable to some hypercholesterolemic patients followed by GPs or specialists. On the other hand, the study has the merits of having been carried out in *real-life settings* in hypercholesterolemic subjects with broad inclusion criteria, which also included a substantial number of patients intolerant to prescription statins, with an overall patient satisfactory compliance.

In conclusion, a nutraceutical combining dry extract of amla fruit, walnut leaves, olive fruit, and RYR powder significantly decreases LDL-C and RC levels in hypercholesterolemic patients in *real-life setting*. The supplementation bears excellent safety and tolerance and is rated as satisfactory, even among patients with statin intolerance.

## Data Availability

The raw data supporting the conclusion of this article will be made available by the authors, without undue reservation.
